# Sternal Foramen Mimicking Gunshot Injury: A Forensic Case Report

**DOI:** 10.7759/cureus.87269

**Published:** 2025-07-04

**Authors:** Arwinder Singh, Yashpal S, Dilip Vaishnav, Kishanth S, Amit Jangid

**Affiliations:** 1 Forensic Medicine and Toxicology, All India Institute of Medical Sciences, Rishikesh, Rishikesh, IND

**Keywords:** congenital anomaly, developmental defect, gunshot wound, skeletal trauma, sternal foramen, sternal foramen vs gunshot wound

## Abstract

The sternum develops from multiple ossification centers, and their incomplete fusion can lead to congenital anomalies like sternal foramina; although typically asymptomatic, these foramina possess significant clinical and forensic relevance due to their potential mimicry of penetrating traumatic injuries such as gunshot wounds. We present a forensic case involving a highly decomposed body, recovered from a submerged vehicle two years post-incident, which exhibited a distinct sternal defect initially suspected as a gunshot wound based on its location and appearance. However, meticulous skeletal examination revealed features consistent with a congenital sternal foramen, including smooth, well-corticated margins and uniform defect dimensions, which allowed for the exclusion of trauma due to the absence of typical gunshot wound characteristics like bevelling, radiating fractures, and bone fragmentation. This case crucially highlights the importance for forensic pathologists to recognize and accurately differentiate anatomical variants like sternal foramina, thereby preventing misinterpretation of the cause and manner of death and ensuring accurate medico-legal conclusions.

## Introduction

The human sternum is a pivotal component of the thoracic cage, which originates from multiple ossification centers that fuse during development [1-4]. Anomalies in this fusion process can lead to developmental defects within the sternal body, such as sternal foremen [1-5]. These congenital defects are often asymptomatic but hold significant clinical relevance, such as the risk of iatrogenic injury during invasive cardiothoracic procedures [1,4,6,7].

In forensic medicine, meticulous examination is necessary for identification of sternal defects. The morphological similarities between a sternal foramen and a penetrating traumatic injury, such as a gunshot wound, can be misleading, especially in cases of advanced decomposition or skeletonization [2,3,8]. Misinterpretation in such scenarios can impact the determination of cause and manner of death, leading to erroneous conclusions in medico-legal investigations [2,3,8]. This article presents a forensic case where such a distinction was critical, followed by a comprehensive review of the differentiating characteristics of sternal foramina and gunshot injuries to the sternum.

## Case presentation

A tragic incident unfolded when a car with a father and son fell into a river. Days later, the son's body was recovered from a downstream dam, and a post-mortem examination was conducted. However, the father's body remained unaccounted for. Two years after the incident, the submerged car was successfully recovered from the riverbed due to a fall in water level. Inside, amidst the advanced stages of decomposition, was the body of the man. Relatives positively identified the car and the deceased based on the clothing still clinging to the remains.

The body was brought for a post-mortem examination, revealing extreme decomposition. While adipocere was observed over the chest area, indicating prolonged immersion in water under specific conditions, the remaining soft tissues and internal viscera were largely unidentifiable. The bones were carefully extracted for detailed assessment.

**Figure 1:**
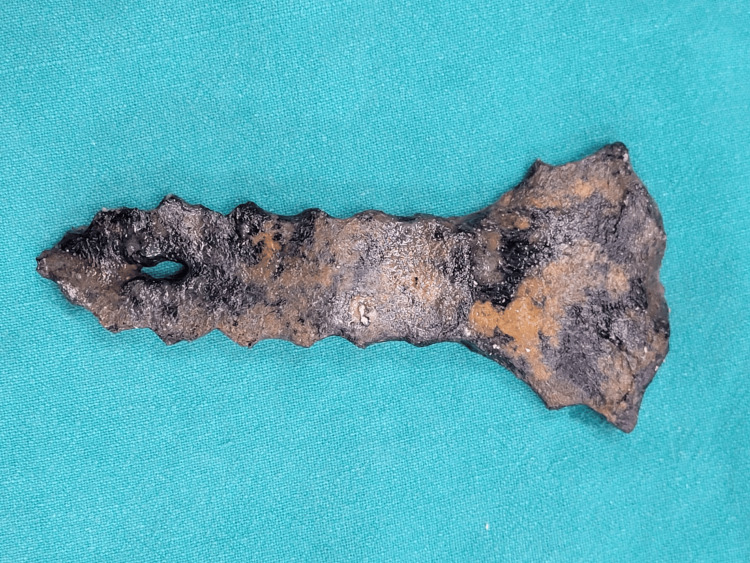
Anterior surface of the recovered sternum. Anterior view of the recovered sternum from the decomposed remains. The distinct oval defect, identified as a sternal foramen, is visible in the lower one-third of the sternal body. Note the well-corticated margins characteristic of a congenital anomaly.

During the examination of the skeletal remains, a noticeable anomaly was discovered: a distinct hole in the lower one-third of the sternum (Figure 1). This finding immediately raised a serious suspicion of a gunshot wound to the chest, suggesting the possibility of a projectile injury that may have precipitated the vehicle's plunge into the river. This potential alternate theory underscored the critical need for a precise differentiation. However, circumstantial evidence, absence of any entry or exit wound, and the bullet in-situ pointed towards the congenital nature of the hole.

**Figure 2:**
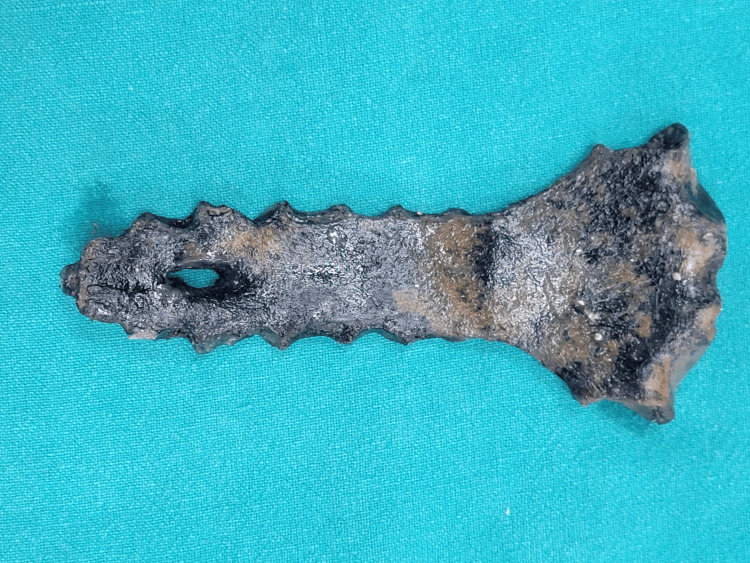
Posterior surface of the recovered sternum. Posterior view of the recovered sternum. The sternal foramen is visible from this aspect, showing the consistent dimensions of the defect across both the anterior and posterior surfaces, further supporting the absence of bevelling.

**Figure 3:**
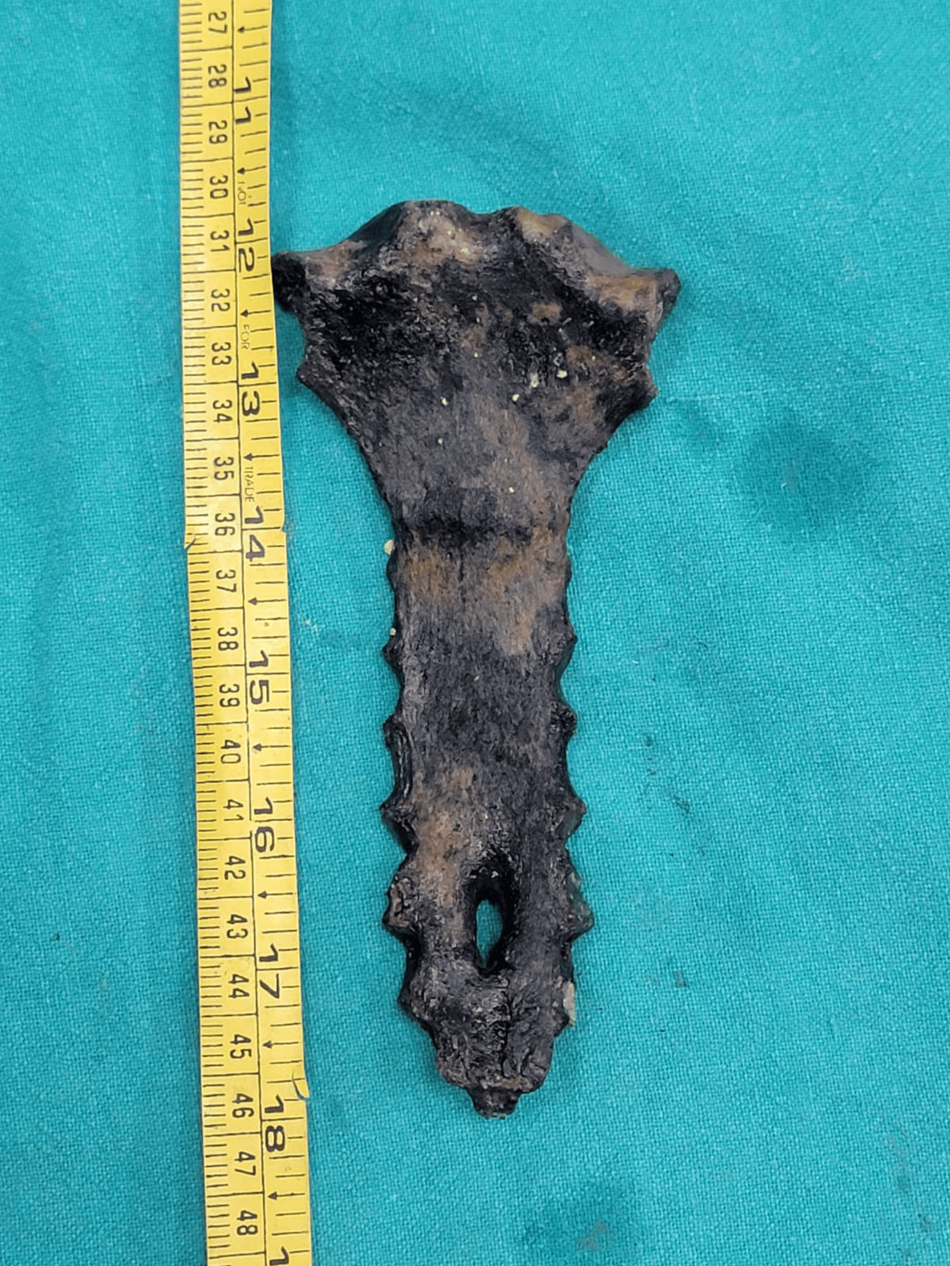
Length measurement of the sternal defect. Recovered sternum with a measuring tape, illustrating the length of the sternal defect. The defect measured 2.2 cm in length at its inner and outer parts, constricting to 1 cm in length at its middle. This view also highlights the surrounding intact bone and the absence of radiating fracture lines.

**Figure 4:**
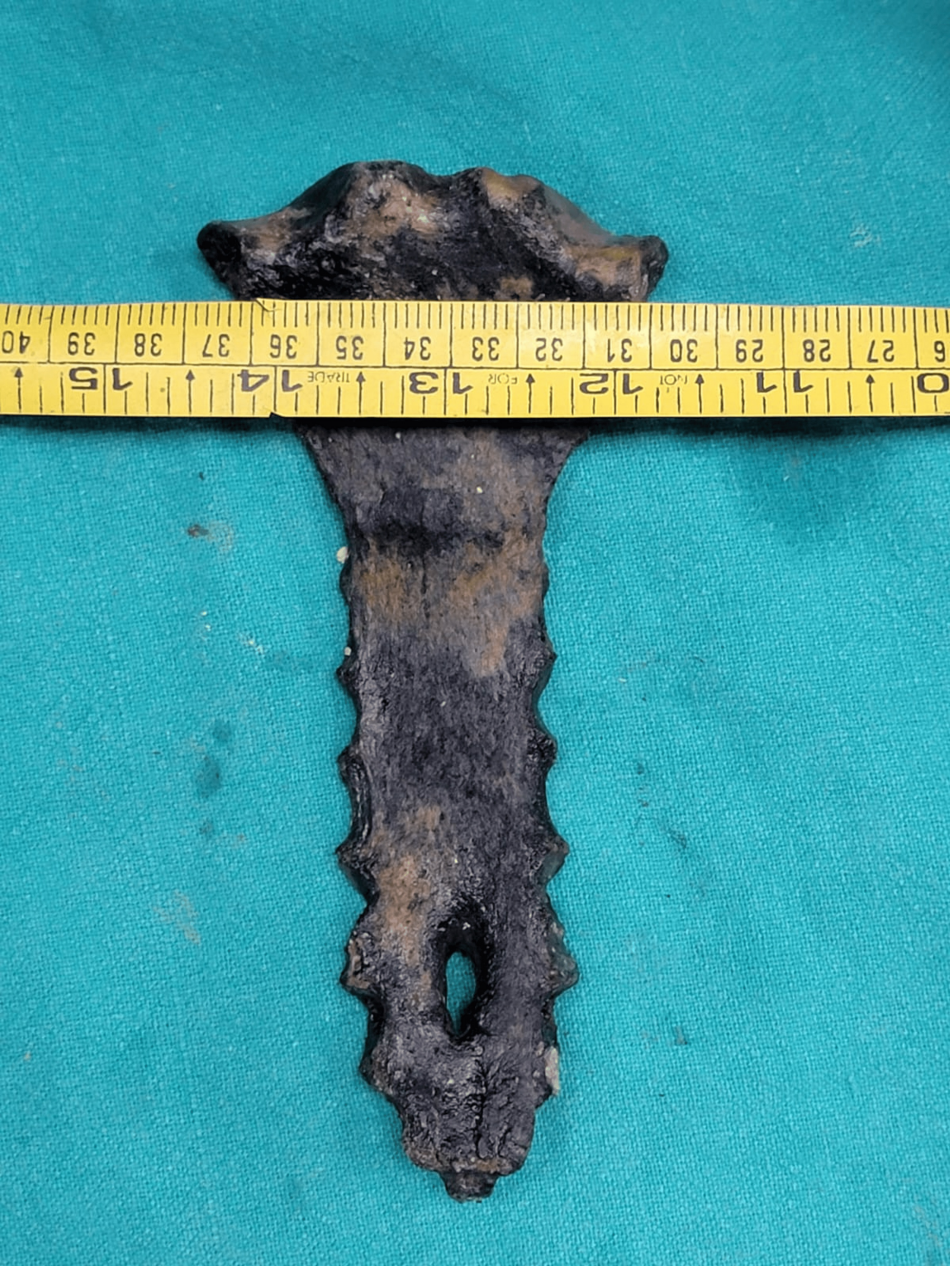
Breadth measurement of the sternal defect. Recovered sternum with a measuring tape, illustrating the breadth of the sternal defect. The defect measured 1 cm in breadth at its inner and outer parts, constricting to 0.4 cm in breadth at its middle. This further demonstrates the uniform nature of the congenital defect.

Detailed examination of the recovered sternum (Figures 1-4) revealed a distinct, oval defect in its lower one-third, measuring 2.2 cm in length and 1 cm in breadth at its inner and outer parts, narrowing to 1 cm in length and 0.4 cm in breadth at its middle.

The margins of the defect were smooth, well-corticated, and regular. The diameter of the defect was consistent across both the anterior and posterior surfaces of the sternum. These findings are consistent with the diagnosis of a sternal foramen. The characteristic features of traumatic impact, such as radiating fracture lines emanating from the defect into the surrounding bone, were absent. Furthermore, bevelling, or the typical conical shape seen in projectile trajectories, was not present. The sternal bone surrounding the anomaly appeared largely intact, lacking the comminution or fragmentation typically associated with a high-energy gunshot wound. While the advanced stage of decomposition precluded soft tissue analysis for gunshot residue, the macroscopic features of the bony defect strongly supported a congenital origin, leading to the conclusion that the anomaly was indeed a sternal foramen rather than a gunshot injury.

## Discussion

Embryological development and ossification

Sternal development is a complex process, beginning with the formation and midline fusion of mesenchymal bars during the sixth to tenth weeks of intra-uterine life, followed by chondrogenesis into a cartilaginous rod. Initial fusion and subsequent segmentation into sternebrae occur in a cranio-caudal direction. Ossification commences around the fifth month of gestation. Manubrium and first two sternebrae form from single centers, and the later sternebrae often develop from paired centers, if bar fusion is not complete. Interestingly, the union of these primary ossification centers progresses in the opposite, caudo-cranial direction, with the xiphoid process ossifying much later. The union of primary ossification centers commences after three years of age, and complete fusion of all sternebrae within the mesosternum is usually observed in individuals over 15 years old [2].

Sternal foramina: Developmental anomalies

Sternal foramina are developmental defects resulting from the incomplete fusion of the sternal segments during ossification. The sternum ossifies from six to eight cartilaginous centers, which fuse sequentially from inferior to superior. Failure of fusion, or partial fusion, can lead to various anomalies, including sternal foramina or sternal clefts [1,3,5].

Sternal foramina are most frequently observed in the lower third of the sternal body, particularly at the junction of the fourth and fifth sternebrae, or occasionally in the xiphoid process or manubrium [3,4]. These congenital defects typically present as round or oval defects, varying in size from a few millimeters to over a centimeter in diameter [3-5]. A defining characteristic of their margins is their smooth, well-corticated, and often everted nature, indicative of a naturally formed anatomical structure, with consistent dimensions across both inner and outer surfaces [1,3-5]. These features were crucial in our case, where the observed sternal defect exhibited precisely these smooth, well-corticated margins and uniform dimensions, directly aligning with the morphological characteristics of a sternal foramen and distinguishing it from a traumatic injury. The reported prevalence of sternal foramina varies across different populations and studies, generally ranging from 4% to 10% in living individuals, though higher in skeletal studies (some reports up to 27.4%), and they are generally more common in males [2-4]. While often asymptomatic, the presence of a sternal foramen is clinically significant as it creates a direct pathway to the mediastinum, carrying a considerable risk of complications during procedures involving sternal puncture, such as bone marrow biopsy or acupuncture, potentially leading to pneumothorax, cardiac tamponade, or injury to great vessels [1,4,6,7].

Gunshot injuries to the sternum: Traumatic insults

Gunshot wounds to the sternum are a result of high-velocity projectile impact, causing significant damage to both bone and surrounding soft tissues. The characteristics of the defect depend on multiple factors, including projectile type, velocity, angle of impact, and range of fire [2,8].

Gunshot wounds are always acquired traumatic lesions, not congenital [9]. Their appearance in bone is distinct: the edges of the bony defect are typically irregular, jagged, and often exhibit radiating fracture lines extending from the primary defect, lacking the smooth cortical bone seen in foramina. A critical feature for determining the direction of projectile travel is bevelling, where the entry wound is wider on the inner aspect of the bone compared to the outer entry point, forming a "cone" effect [9]. The impact also commonly causes significant bone fragmentation (comminution), leading to multiple small pieces of bone (shrapnel) [10]. If the projectile exits the sternum, the exit wound is generally larger and more irregular, exhibiting external bevelling [9]. In our case, the sternal defect conspicuously lacked these features; there were no irregular, jagged edges, no radiating fracture lines, no bevelling indicating projectile trajectory, and no evidence of comminution or fragmentation, which strongly argued against a gunshot injury. Associated soft tissue damage is also characteristic of gunshot wounds, including an abrasion collar around the skin entry wound, and for close-range shots, soot and unburnt/partially burnt gunpowder particles (stippling or tattooing) [11]. Extensive tissue damage, hemorrhage, and disruption to underlying muscles and vital organs (heart, lungs, major blood vessels) are almost invariably present [9]. Radiographic imaging or dissection may also reveal retained bullet fragments within the sternal bone or surrounding tissues [9]. Despite the advanced stage of decomposition in our case, the absence of any remnants suggesting an abrasion collar, soot, or stippling, combined with the lack of any projectile fragments within or near the sternal defect, further supported the exclusion of a gunshot wound.

Differentiating sternal foramina from gunshot injuries: A forensic perspective

The ability to accurately distinguish between a sternal foramen and a gunshot injury (Table 1) is paramount in forensic pathology and anthropology, especially in challenging cases like the one presented [2,3,8].

**Table 1:** Key differentiating features between the sternal foramen and gunshot injury. This table outlines the key characteristics that distinguish a sternal foramen (a congenital or developmental anomaly) from a sternal defect caused by a gunshot injury (an acquired traumatic event) [1,2,9,11].

Feature	Sternal foramen	Gunshot injury
Origin	Congenital/developmental	Acquired (traumatic)
Margins	Smooth, rounded, well-corticated, often everted	Irregular, jagged, fractured, and absent cortical bone
Bevelling	Absent (same dimensions on both sides)	Present (internal for entry, external for exit)
Radiating fractures	Absent	Often present, extending from the defect
Bone fragmentation	Absent	Often present (comminution)
Associated soft tissue damage	Absent	Almost always present (lacerations, hemorrhage, charring)
Gunshot residue	Absent	May be present (soot, stippling) at entry
Underlying organ damage	Unrelated (unless iatrogenic complication)	Common and severe (heart, lung, vessels)
Radiological appearance	Smooth, corticated border, uniform defect	Irregular, fragmented, often with associated foreign bodies

## Conclusions

This case illustrates the challenges faced in forensic medicine, particularly when dealing with highly decomposed remains. The initial suspicion of a gunshot injury due to the sternal defect underscores the critical need for a precise differential diagnosis. Through meticulous examination, focusing on the definitive morphological characteristics of the sternal defect -- namely the smooth, corticated margins, the absence of beveling, and the lack of associated traumatic indicators on the skeletal remains and surrounding minimal tissues -- we were able to conclude that the "hole" was indeed a sternal foramen, a congenital anomaly, and not a gunshot wound.

This determination had profound implications, allowing investigators to correctly focus on drowning as the cause of death in the context of the vehicle's plunge into the river. This case reinforces the absolute necessity for forensic experts to possess a deep understanding of anatomical variations and their differentiating features from traumatic injuries, ensuring accuracy in medico-legal investigations and upholding the integrity of justice.

## References

[1] Choi PJ, Iwanaga J, Tubbs RS (2017). A comprehensive review of the sternal foramina and its clinical significance. Cureus.

[2] Cooper PD, Stewart JH, McCormick WF (1988). Development and morphology of the sternal foramen. Am J Forensic Med Pathol.

[3] Gkantsinikoudis N, Chaniotakis C, Gkasdaris G, Georgiou N, Kapetanakis S (2017). Morphological approach of the sternal foramen: an anatomic study and a short review of the literature. Folia Morphol (Warsz).

[4] Pasieka P, Pasieka PM, Komosa A, Barnowska A, Pękala J, Malinowski K, Tomaszewski K (2023). Prevalence and morphometry of sternal and xiphoid foramen: a meta-analysis on 16,666 subjects. Surg Radiol Anat.

[5] Lema AS (2023). Anatomical variations of the sternum: sternal foramen and variant xiphoid morphology in dried adult human sternum in Ethiopia. F1000Res.

[6] Stark P (1985). Midline sternal foramen: CT demonstration. J Comput Assist Tomogr.

[7] Yekeler E, Tunaci M, Tunaci A, Dursun M, Acunas G (2006). Frequency of sternal variations and anomalies evaluated by MDCT. AJR Am J Roentgenol.

[8] McCormick WF, Flournoy LE, Rogers NL, Ross AH (1998). An unusual case of multiple mesosternal foramina. J Forensic Sci.

[9] Di Maio VJM (1999). Gunshot Wounds: Practical Aspects of Firearms, Ballistics, and Forensic Techniques.

[10] Humphrey C, Henneberg M (2017). Anthropological analysis of projectile trauma to the bony regions of the trunk. Anthropol Rev.

[11] Shrestha R, Kanchan T, Krishan K (2025). Gunshot wounds forensic pathology. StatPearls (Internet).

